# Investigation of the Molecular Evolution of Treg Suppression Mechanisms Indicates a Convergent Origin

**DOI:** 10.3390/cimb45010042

**Published:** 2023-01-09

**Authors:** Suniti Bhaumik, Marzena Łazarczyk, Norwin Kubick, Pavel Klimovich, Agata Gurba, Justyna Paszkiewicz, Patrycja Teodorowicz, Tomasz Kocki, Jarosław Olav Horbańczuk, Gina Manda, Mariusz Sacharczuk, Michel-Edwar Mickael

**Affiliations:** 1 Department of Pathology, University of Alabama at Birmingham, 845-19th St. South, Birmingham, AL 35294-2170, USA; 2 Department of Experimental Genomics, Institute of Animal Biotechnology and Genetics, Polish Academy of Science, Postępu 36A, 05-552 Jastrzebiec, Poland; 3 Department of Biology, Institute of Plant Science and Microbiology, Univeristy of Hamburg, Ohnhorststr. 18, 22609 Hamburg, Germany; 4 Department of Immunology, PM Forskningscentreum, 17854 Ekerö, Sweden; 5 Department of Pharmacodynamics, Faculty of Pharmacy, Warsaw Medical University, l Banacha 1, 02-697 Warsaw, Poland; 6 Department of Health, John Paul II University of Applied Sciences in Biala Podlaska, Sidorska 95/97, 21-500 Biała Podlaska, Poland; 7 Department of Experimental and Clinical Pharmacology, Medical University of Lublin, Jaczewskiego 8b, 20-090 Lublin, Poland; 8 Institute of Genetics and Animal Breeding, Polish Academy of Sciences, 05-552 Jastrzębiec, Poland; 9 Radiobiology Laboratory, Victor Babes National Institute of Pathology, 99-101 Splaiul Independentei, 050096 Bucharest, Romania

**Keywords:** Tregs, evolution, ai

## Abstract

Regulatory T cell (Treg) suppression of conventional T cells is a central mechanism that ensures immune system homeostasis. The exact time point of Treg emergence is still disputed. Furthermore, the time of Treg-mediated suppression mechanisms’ emergence has not been identified. It is not yet known whether Treg suppression mechanisms diverged from a single pathway or converged from several sources. We investigated the evolutionary history of Treg suppression pathways using various phylogenetic analysis tools. To ensure the conservation of function for investigated proteins, we augmented our study using nonhomology-based methods to predict protein functions among various investigated species and mined the literature for experimental evidence of functional convergence. Our results indicate that a minority of Treg suppressor mechanisms could be homologs of ancient conserved pathways. For example, CD73, an enzymatic pathway known to play an essential role in invertebrates, is highly conserved between invertebrates and vertebrates, with no evidence of positive selection (w = 0.48, *p*-value < 0.00001). Our findings indicate that Tregs utilize homologs of proteins that diverged in early vertebrates. However, our findings do not exclude the possibility of a more evolutionary pattern following the duplication degeneration–complementation (DDC) model. Ancestral sequence reconstruction showed that Treg suppression mechanism proteins do not belong to one family; rather, their emergence seems to follow a convergent evolutionary pattern.

## 1. Introduction 

The mechanisms of Treg suppressor function can be categorized into two main pathways: (i) direct pathways, through suppression of CD4^+^ CD25^−^ T cells, and (ii) indirect pathways, through inhibiting antigen-presenting dendritic cells (DCs). Direct pathways of Treg suppression include suppression of CD4^+^ CD25^−^ T cells through the release of cytokines such as TGFβ, IL10, and IL35. However, the role of TGFβ in Treg-mediated suppression remains complicated [1,2]. IL10 produced by Tregs has been shown to possess an inhibitory effect. CD25^+^CD45RB^low^ CD4+ T cells from *Il10*^−/−^ mice were unable to inhibit colitis, and *Il10r^−/−^* mice did not develop autoimmunity [3,4]. The importance of IL35 (IL27β and IL12α) in the Treg suppression function is underlined by the low inhibitory capacity of Il35^−/−^ Tregs [5]. The second mechanism that Tregs can employ is the consumption of critical cytokines needed by conventional T cells for survival, such as IL2 [6]. Thirdly, Tregs can induce apoptosis using TRAIL, CD3, CD46, CD25, or BIM pathways. Furthermore, it has been shown that Tregs use inducible cAMP early repressor (ICER) to suppress CD25+ CD4+ T cells NFAT pathway [7]. Additionally, Tregs have been shown to regulate calcium pathways needed for T cell differentiation through a calcineurin-dependent pathway. Recently, it was shown that an additional mechanism used by Tregs to control suppressor function in colonic Tregs is through the Th17 specific transcription factor RORγt [8]. There are various indirect mechanisms by which Tregs suppress conventional T cells including: CTLA4 (cytotoxic T lymphocyte-associated protein 4)-dependent pathway, where it was shown that CTLA4 expressed on Tregs competes with CD28 for its ligands (i.e., CD80/CD86), expressed by DCs to restrict CD28 co-stimulation of other T cells [9]. A transmembrane glycoprotein receptor, Neuropilin-1 (Nrp1) expressed on Tregs was shown to enhance Treg suppressor function by extending the interactions between Tregs and DCs, thus limiting antigen exposure to conventional T cells [10]. LAG3 (lymphocyte-activation protein-3) expressed on Treg cells bound to DCs was demonstrated to inhibit DC maturation and immunostimulatory capacity [11]. Additionally, A20, a ubiquitin editing enzyme, is a negative regulator of TNFR and Toll-like receptor (TLR) signaling in antigen-presenting DCs [12]. Notably, the interaction between Tregs and DCs is multidimensional, as it has been shown that Tregs cannot suppress DCs which are either CD40 “licensed” or have achieved a superior stimulation state through TLR activation [13]. Thus, Treg inhibition of CD4+ CD25+ T cells is based on the complex interaction between several genetic pathways. 

The evolutionary history of the regulatory mechanisms utilized by Tregs has not yet been unveiled. T cells first appeared in jawed vertebrates [14,15]. The exact time Tregs and Treg-like cells emerged in lower vertebrates (bony fish and lampreys) is still a point of dispute [16]. It is still not known if the suppression pathways used by Tregs constitute a vertebrate innovation or whether they were acquired from invertebrates. Even more, the necessity of Tregs in lower vertebrates is being questioned. One side of the argument suggests that Treg suppression mechanisms are universal, suggesting that they could have evolved from one initial abstract mechanism. The other side of the argument suggests that their evolution was convergent, with multiple local mechanisms appearing in various organisms [14]. Inferring neo-function emergence is not a trivial task. Currently, two competing models predict the rise of novel functions: (i) the conventional model; and (ii) the duplication-degeneration–complementation model (DDC). In the conventional model, it is assumed that neo-functions arise after duplication, where one copy preserves the original function, and the mutation in the other copy drives the neo-functionalization. According to the DDC model, a gene’s function in a specific species does not necessarily appear when this gene originates. The DDC model proposes the possibility that the function could have been performed by an ancestral pre-duplicative gene in an ancestral species, in a sub-functionalization manner. Thus, shared homology or orthology, or the lack of it, may not be sufficient to deduce or exclude conservation of function. Another technical obstacle facing the elucidation of the Treg suppression function is the lack of specific markers that guarantee the conservation of function among species. The presence of a specific marker in a species does not necessarily imply it portrays a given function or a specific cell type in another species. For instance, invertebrates, such as Amphioxus (cephalochordates) possess all the genes necessary for the neural crest cells, but they lack neural crest cells. Moreover, there is a debate about the origin of mammalian Treg suppressive mechanisms. Furthermore, how lampreys mediate control over their immune-like cells is still unknown [15]. Lampreys are known to possess two types of T-cell-like cells, which exist in the blood and are affected by cytokines (i.e., VLRA and VLRC) [17]. The mechanisms by which the functions of these immune-like cells in lampreys are regulated are yet to be found. Taken together, these observations suggest there is a need for a better understanding of the origin and evolutionary history of the suppressive mechanisms of Tregs. 

To increase our investigation’s predictive power, we utilized both homology-based and non-homology-based procedures in our quest to investigate the evolution of Treg suppressor pathways. First, we conducted a phylogenetic analysis. As the genes controlled by Treg suppression mechanisms can amount to hundreds of genes, we selected representative proteins from major Treg suppression pathways based on past reviews [18,19,20,21] (Table 1). We downloaded the protein sequences related to Treg suppression mechanisms from humans’ GEO PubMed protein repository. Using Blastp, we identified homologs for these sequences in 12 species and orders. To support our findings, we used statistical inference-based methods that do not use homology for the deduction of functional conservation. DeepGO uses two methods to predict protein function, the first method learns features from protein sequence, without making assumptions on homology. The second method learns representations of proteins based on their location in an interaction network [22]. We performed systematic literature mining to support our predictions. After that, we reconstructed the ancestor sequences for known Treg suppressors and investigated the evolutionary selection of these suppressors using PAML. Our findings demonstrate that a minority of the identified conserved suppression-mechanism-associated genes seem to perform similar functions in vertebrates and invertebrates. We found that numerous suppressive mechanisms that exist in vertebrates seem to have formed in concurrency with Treg emergence. However, these findings do not exclude the hypothesis that similar suppression functions could have appeared earlier. Our results support the argument Treg cells suppressive pathways do not seem to be unique and that they appeared through different convergent mechanisms starting from early invertebrates. We found several suppressor mechanisms in lampreys, which suggests that lampreys could possess FoxP3 suppressor cells. Taken together, our investigation paints Tregs as an “evolutionary-wise” suppressive group of cells that could have accommodated evolutionary genomic innovations and, in some instances, repurposed more ancient ones. 

## 2. Methods 

### 2.1. Database Search 

Here we have focused our investigation on the suppression mechanisms utilized by Tregs as well as Treg markers. We employed protein sequence alignment to explore the relatively long evolutionary history of Tregs and their regulatory mechanisms. We utilized the non-redundant protein NCBI protein database (last updated 5 March 2022) with a total number of sequences of 467,645,306 [23,24]. This database is a collection of carefully curated records from GenBank, RefSeq, and TPA. Using this database, we investigated the presence of known humans *Homo sapiens* (NCBI: txid 9606) Treg markers, as well as identifying Treg suppression pathways in Rodentia (rodent) (NCBI:txid9989), Monotremata (egg-laying mammals) (NCBI:txid9255), Metatheria (marsupials) (NCBI:txid9263), Sauria (Diapsida) (NCBI:txid32561), Actinopterygii (ray-finned fishes) (NCBI:txid7898), Anura Salientia (NCBI:txid8342), Petromyzontiformes lampreys (NCBI:txid7745), Tunicata (tunicates) (NCBI:txid7712), Arthropoda (arthropods) (NCBI:txid6656), Nematoda (roundworms) (NCBI:txid6231), Spiralia (NCBI:txid2697495), Cnidaria (cnidarians) (NCBI:txid6073), Placozoa (placozoans), (NCBI:txid10226), and Porifera (sponges) covering 700 million years using BLASTP (Table 1). To ensure the accuracy of our analysis, in cases where multiple isoforms were identified, only the longest transcript was utilized. We used a threshold of E values of ≤1 × 10^−10^. For any candidate protein to be accepted, it had to have the same conserved domains known in the human query protein [25,26,27]. Furthermore, we use back blastp to ensure that the results are consistent by blasting the identified candidate protein sequence against the human protein sequence database. 

### 2.2. Alignment and Phylogenetic Analysis 

The phylogenetic investigation was performed in two phases. First, protein sequences for each identified pathway were aligned using ClustalW in SEAVIEW (Table 1). After that, we employed PHYML implemented in Seaview, with five random starting trees to generate the final tree [25,28]. 

### 2.3. Ancestral Sequence Reconstruction (ASR) 

We utilized the maximum likelihood method to predict the ancestral sequence of each of the proteins investigated. To do that, we utilized the ASR algorithm in MEGA6. We then used the predicted sequence to unravel the nearest earlier diverging organism. We used a Blastp threshold E-value of less than e^−10^ to ensure the accuracy of our identification. Finally, we employed SplitsTree with default settings and bootstrap value of 100 to construct the evolutionary network for ancestral sequences [29]. 

### 2.4. HHsearch 

The HHsearch method was applied to inspect the evolutionary history of Tregs as well as their suppression mechanisms. To ensure the validity of our predictions, we only accepted proteins that appeared in the organism that diverged before the emergence of the most ancient members of the inspected protein [30].

### 2.5. Non-Homology Functional Prediction 

In order to support our results, we conducted functional prediction for each of the investigated proteins using multiple non-homology-based methods. We used DeepGO, which employs two neural networks and gene network connections to predict protein functions [22]. We also used Pannzer, which utilizes a weighted K-nearest neighbor classifier to infer the function of a given protein sequence [31]. We also employed deep learning embedding (GO and secondary structure) using the PredictProtein method [32]. Additionally, we used Argot2, which primarily applies semantic similarity for prediction.

### 2.6. Positive Selection 

We employed the maximum likelihood algorithm in Phylogenetic Analysis by Maximum Likelihood (PAML) to detect Treg suppressor pathways that have experienced positive selection. In the first phase, we utilized respective complementary DNAs (cDNAs) constructed through the back-translation function from the EMBOSS server (https://www.ebi.ac.uk/Tools/st/emboss_backtranseq/ accessed on 9 September 2021). After that, we prompted the CODEML PAML v4.4 program to assess global and branch selection by calculating the substitution rate ratio (ω) computed as the ratio of nonsynonymous (dN) to synonymous (dS) mutations [33].

## 3. Results 

**Figure 1:**
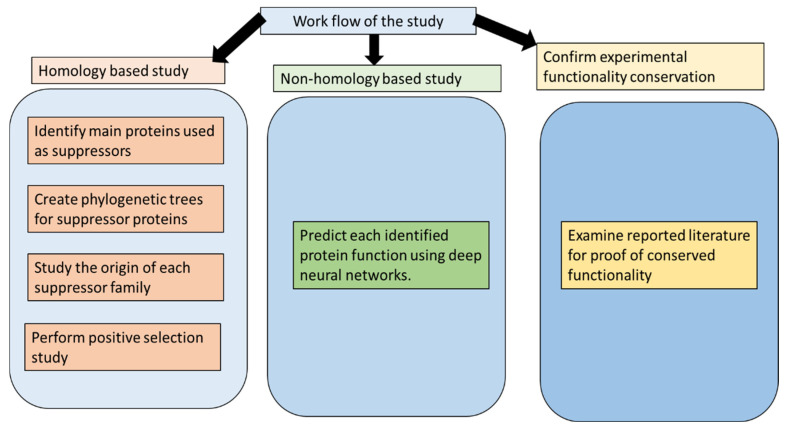
Workflow of the study. We approached the problem of investigating conserved Treg suppression pathways through three different pathways: (i) Homology based, in which we followed classical phylogenetic analysis procedures. (ii) Non-homology-based approach, where we utilized artificial intelligence to predict the function of the identified proteins. (iii) In order to increase the accuracy of our estimations, we compared our predictions with published reports that experimentally validated functional conservation of Treg suppressor proteins among different species.

We used a comprehensive workflow to investigate the evolution of various groups of the main proteins involved in Treg suppressive pathways (Figure 1 and Table 1). It is important to note that, according to the DDC model, non-conservation of function does not necessarily imply that the organisms that lack a specific gene did not have an ancestral gene that was performing the same function. Thus, in our analysis, we only focus on the direct conservation of sequences (homology and non-homology based) that are related to the conservation of functions. Our workflow consisted of three main stages: (i) Phylogenetic analysis: in this stage, we identified some of the main proteins used by Tregs as suppressors. Then, we created phylogenetic trees, estimated the origin, and investigated positive selection for each suppressive family. (ii) Additionally, using neural networks, we predicted the function of each identified protein. (iii) We examined the literature to investigate the reported incidence of functional conservation. 

**Figure 2:**
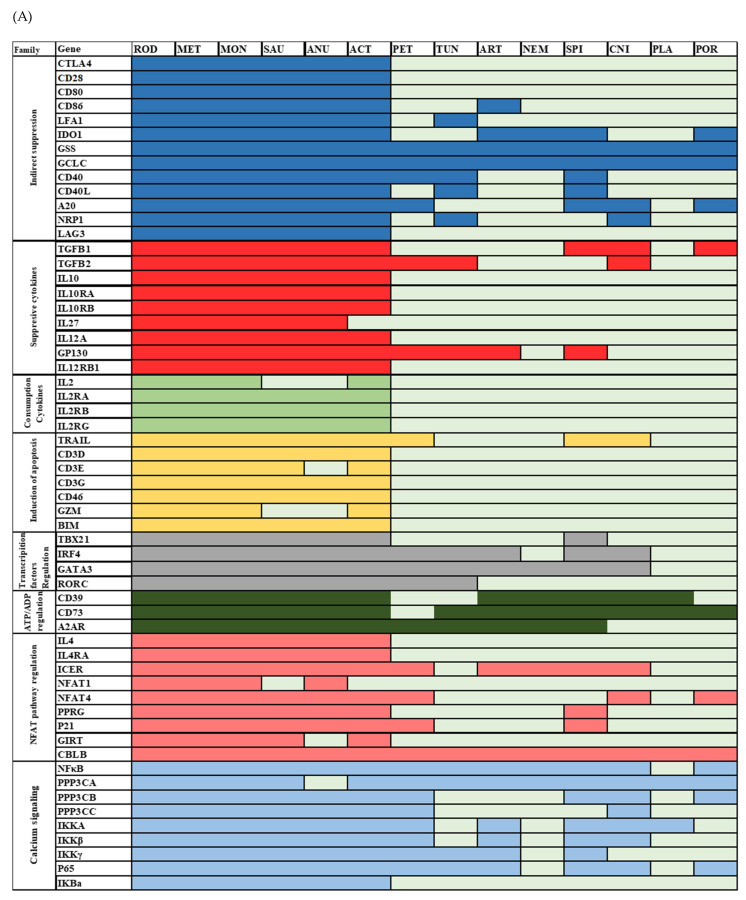

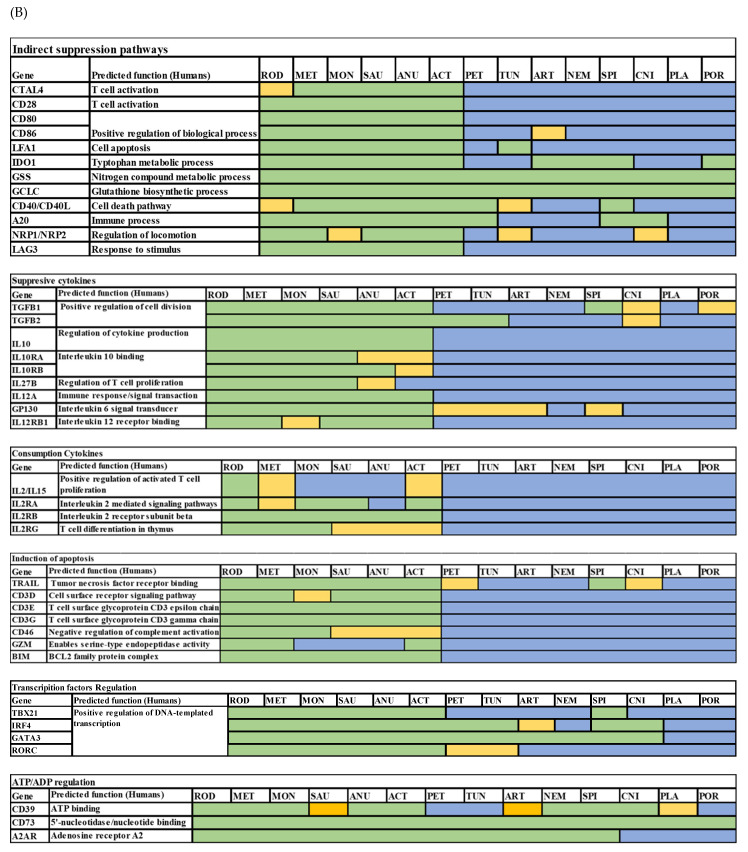

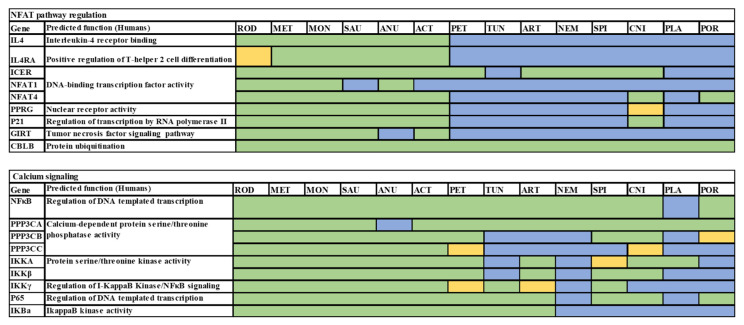
Evolutionary history of Treg-mediated suppression pathways. (**A**) Phylogenetic analysis of Treg pathway (homology based). Each family identified in Table 1 is shown with a different color code. (**B**) Non-homology analysis. Non-homology-based analysis does not assume that homology is the basis of functional conservation. Proteins that have similar predicted functions to their human counterparts are shown in green, while those predicted to perform alternative functions are depicted in orange. Proteins that were deemed to be missing from a given proteome are shown in light blue. The following abbreviations are used: Rodentia (ROD), Monotremata (MON), Metatheria (MET), Sauria (SAU), Actinopterygii (ACT), Anura Salientia (ANU), Petromyzontiformes (PET), Tunicata (TUN), Arthropoda (ART), Nematoda (NEM), Spiralia (SPI), Cnidaria (CNI), Placozoa (PLA), and Porifera (POR). Overall, there is agreement between phylogenetic-based prediction and functional prediction using artificial intelligence/machine learning methods. Although Treg cells seem to have first appeared in jawed vertebrates, they employed various more ancient pathways. For example, TGFβ1, NFAT, NFκB first appeared in Porifera. Various suppression pathway components appeared in lampreys, including the PPP3C family, as well as TGFβ.

**Figure 3:**
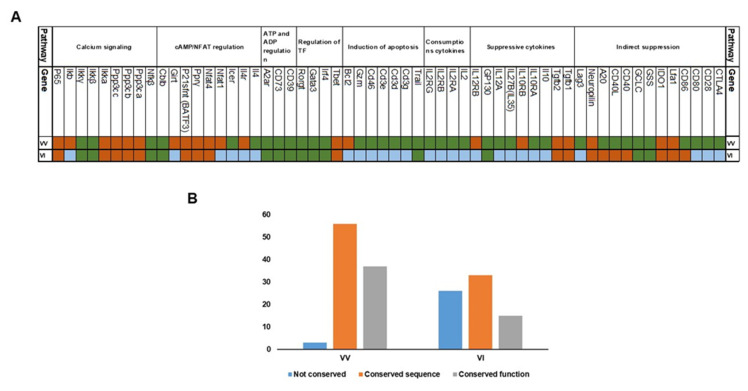
Analysis of literature reporting functional conservation of Treg suppressor mechanisms. (**A**) We compared 60 proteins for reported conservation between vertebrate–vertebrate (VV) and between vertebrates and invertebrates (VI) (green is conserved, red is non-conserved). (**B**) Percentages of functional conservation by category.

Evolutionary analysis of Treg suppressor mechanisms revealed that a minority of Tregs suppressive mechanisms could be “*directly*” related to conserved suppressive mechanisms in invertebrates. We found that, with the exception of TGFβ, none of the cytokines produced by Tregs to directly inhibit conventional T cells was conserved in invertebrates (Figure 2A,B). The suppressive cytokines utilized by Tregs include TGFβ (including TGFβ1 and TGFβ2), IL35 and IL10. Our results indicate that TGFβ homologs are ancient. TGFβ1 first appeared in sponges, while TGFβ2 first appeared in Cnidaria (Figure 2). In agreement with [26], we found that IL10, as well as IL10Rα and IL10Rβ, diverged during the emergence of Actinoptyregii. IL-35, a dimeric protein of IL27β and IL12α chains, is an anti-inflammatory cytokine [34]. Its receptor is composed of gp130 and IL12Rβ chains. The gp130 chain first appeared in Spiralia, while IL27β, IL12α, and IL12Rβ proteins appeared first in fish (Figure 2). Remarkably, we found that IL2, and its three receptors IL2Rα, IL2Rβ, and IL2Rγ, first diverged in fish. Notably, we found reported evidence of functional convergence for IL10, IL10RA, IL12A, IL35 and GP130 in vertebrates. Conversely, only a primitive GP130 homolog was found in invertebrates with some functional similarities between vertebrates and invertebrates (Figure 3).

Similar to cytokines used by Tregs, Treg-mediated induction of apoptosis pathway lacks homologs that emerged before vertebrates, with the exception of TRAIL (TNF-related apoptosis-inducing ligand). We were able to locate the TRAIL gene in Lampreys and invertebrates (including Mollusca and Cnidarian). Notably, the Trail *Drosophila* homolog (i.e., *Eiger*) has been reported to induce apoptosis, thus supporting the functional conservation hypothesis (Figure 2 and Figure 3). The CD3γ, CD3δ, and CD3ε chains form the CD3 part of the TCR complex. According to our results, their first diverging homologs emerged during the emergence of fish, where they are functionally conserved (Figure 3). Similarly, the costimulatory molecule CD46, which has been shown to enhance Treg activation, was functionally conserved in bony fish (Figure 2 and Figure 3). Once activated, Tregs have been reported to induce apoptosis in conventional T cells through granzyme A (GZMA)-mediated pathway [23]. We found that GZMA and BIM (Bcl2-like protein 11) have diverged in fish. We found reported evidence for functional conservation of mediated induction of apoptosis between mammalian and fish T cells except for the case of BIM (BCL2L11) (Figure 3, Table 2). 

Tregs mediate indirect changes in the transcription factors of different T helper cell subsets. T-bet, the transcription factor of Th1 cells, first diverged during Molluscan evolution, with some functional conservation in ectoderms (Figure 2 and Figure 3). The transcription factor IRF4 is critical for the function of Th2 and Th17 cells, and we observed that it first appeared in Cnidarian (Figure 2) [35]. IRF4 function seems to be conserved between mammalian and fish T cells, with some conserved function in invertebrates (Figure 3). Gata3, a transcription factor of Th2 cells appeared in all investigated genera starting from Cnidaria and seems to be conserved outside the realm of T cells (Figure 2 and Figure 3) [36]. The transcription factor RORC first appeared in Tunicate (Figure 2 and Figure 3), albeit without proof of ancient conserved functionalization. Notably, FoxP3, which is the main transcription factor of Tregs, is known to have diverged in vertebrates. Taken together, our results indicate that Tregs acquired mechanisms that can regulate the master TF of T helper cells. These TF seem to have emerged prior to Treg appearance in bony fish. However, there might not have been functional conservation between these TF vertebrate homologs and their invertebrate counterparts.

Another route by which Tregs suppress conventional T cells is through the hydrolysis of extracellular ATP to ADP or AMP, utilizing the enzyme CD39. We found that CD39 diverged during the emergence of Placozoa, with proof of some functional conservation [37]. However, our AI-based analysis did not confirm functional conservation in placozoa. Notably, a CD39 homolog gene was also found in Drosophila [37]. CD73 was also shown to inhibit conventional T cells through degrading AMP to adenosine. We identified CD73 homologs in all genera investigated, from Sponges to Humans except Lampreys (Figure 2). CD73s expressed in vertebrates appear to share functional similarities with their invertebrate counterparts [38]. Interestingly, adenosine receptors seem to have diverged during Cnidarians’ emergence, with some functional conservation (Figure 3) [37].

Functional conservation validation for the Inducible cAMP Early Repressor (ICER) pathway is still to be achieved on the level of both vertebrates and invertebrates, with the exception of Cblb (Figure 2 and Figure 3). Tregs suppress conventional T cells by augmenting ICER to suppress IL4. We found that ICER homologs were expressed in Cnidarian (Figure 2), while IL4 and IL4Rα first appeared during the emergence of Actinopterygii. However, we could not identify previous reports that confirmed their functional conservation. Another ICER-mediated suppression pathway is through NFAT. NFAT forms inhibitory complexes with ICER or similar transcriptional repressors such as Peroxisome proliferating receptor (PPRγ) and basic leucine zipper protein p21SNFT. These complexes attach to cytokine promoters to inhibit their function. We were able to identify NFAT4 in sponges, while NFAT1 seems to have recently diverged during the emergence of Xenopus. The emergences of PPRγ and p21SNFT were more ancient, and they both first appeared in Spiralia (Figure 2). Interestingly, ICER induction in suppressed T cells was GITR dependent. GITR (Glucocorticoid-Induced Tumor Necrosis Factor-Related Receptor) first appeared in fish (Figure 2) [38]. Additionally, it is notable to mention that Cblb^−/−^ T cells are less sensitive to suppression by Tregs. Interestingly, Cblb (Casitas B-lineage lymphoma protooncogene-b) an E3 ligase and known as one of the most important gate keepers of immune activation, was identified in all species from sponges to humans (Figure 2).

**Figure 4:**
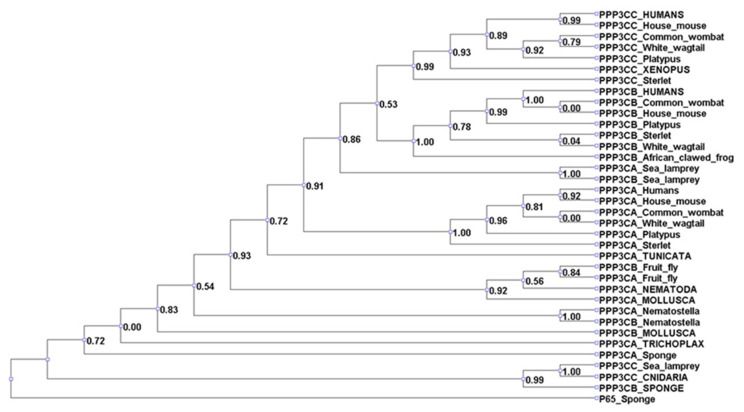
PPP phylogenetic analysis. Our analysis of the PPP family shows that it has an ancient history, as it first emerged during sponge divergence. Interestingly, Tregs inhibit the calcineurin pathway to halt the differentiation of conventional T cells.

An additional aspect of Treg-mediated regulation of conventional T cells is the suppression of calcium signaling. Calcineurin, a ubiquitous serine/threonine protein phosphatase, has been suggested to inhibit NFκB, through an IKK-mediated pathway. Calcineurin has three family members, namely PPP3CA, PPP3CB, and PPP3CC. Calcineurin evolution is diverse, with the PPP3CA and PPP3CB isoforms emerging as early as sponges, while the PPP3CC isoform first appeared in Mollusca (Figure 2, Figure 3 and Figure 4). Remarkably, our results indicate that NF-κB is as ancient as sponges. The IKK complex, required for NFκB activation, consists of three subunits, and all of them emerged during invertebrates’ divergence, whereas p65 first appeared in Porifera. We only found evidence of functional conservation for NFκB and two units of the IKK complex, namely, IKKβ and IKKγ. 

We identified various homologs of proteins used by Treg to indirectly suppress CD25^−^ CD4+ T cells in species that emerged before Tregs emergence in jawed vertebrates. Mammalian CTLA4 performs its function through four different pathways: (i) CTLA4 expressed on Tregs compete with CD28 expressed on conventional T cells through binding to CD80/CD86 expressed on APC [39]; (ii) a line of action that is dependent on LFA1-ICAM1 interaction [40]; LFA1 (Lymphocyte function associated antigen 1) is a heterodimer of two proteins, ITGAL and ITGB2 [41]; (iii) CTLA4 can increase the expression of IDO (Indoleamine 2,3-dioxygenase) in APC to starve T cells [21]; and (iv) CTLA4 can reduce the expression of GCLC (Glutamate-cysteine ligase catalytic subunit) and GSS (Glutathione synthase) to form an unfavorable redox environment for T cells [21]. We could not find any homologs for CTLA4, CD80 and CD28 beyond vertebrates. Interestingly, fish and mammalian CTLA4, and CD80 seem to be functionally conserved (Figure 2 and Figure 3). We found three putative homologs of CD86 in Arthropoda, namely *Ostrinia furnacalis*, *Vanessa tameamea*, and *Pieris rapae* (XP-028167410.1, XP-026493316.1, and XP-022129956.1). The ITGAL homolog was identified in Tunicates, while IDO, as well as GSS and GCLC homologs, were localized in sponges (Figure 2). However, while there is evidence for functional conservation for both GSS and GCLC between invertebrates and vertebrates, no evidence for functional conservation for LFA1 or IDO exists. We found that the CD40-CD40L pathway is functionally conserved in fish (Figure 2 and Figure 3). We found homologs for both CD40 and CD40L in Mollusca, albeit without evidence for conservation of function pre-vertebrate emergence (Figure 3). Moreover, we found that A20 homologs first appeared during the emergence of sponges, while NRP-1 first appeared in Cnidaria. However, there is no reported evidence of functional conservation. LAG3 (Lymphocyte-activation gene 3) seems to have diverged during fish emergence, with reported functional conservation between fish and mammalian homologs (Figure 3).

### 3.1. Analysis of Treg Suppressor Markers Reveals Multiple Origins of Suppression Mechanisms 

We identified the main suppressors used by Tregs to be TGFβ, TRAIL, GZM, CD39, CD73, ICER, NFAT, PPRγ, P21SNFT, GITR, and Cbl-b, as well as indirectly through CTLA4, CD40, CD40L, A20, Neuropilin1, and LAG3. Investigating the structure of these identified suppressors revealed that they belonged to various families, namely (i) the immunoglobulin superfamily, which included CTLA4 and LAG3, (ii) TNF superfamilies, its associates and their receptors, which included CD40L (TNFSF5), CD40, A20 (TNFAIP3), TRAIL (TNFSF10) and GITR, (iii) Neuropilins, (iv) the TGFβ superfamily, (v) Granzymes, (vi) the E-NTPDase family of ectonucleotidases, (vii) Ecto-5’-nucleotidase, (viii) bZIP transcription factor domain-containing protein, (ix) NFATs, (x) 5-hydroxy eicosatetraenoic acid and the 5-oxo-eicosatetraenoic acid family, (xi) the basic leucine zipper transcriptional factor ATF-like family, and (xii) the ubiquitin ligase family (Table 3). 

**Figure 5:**
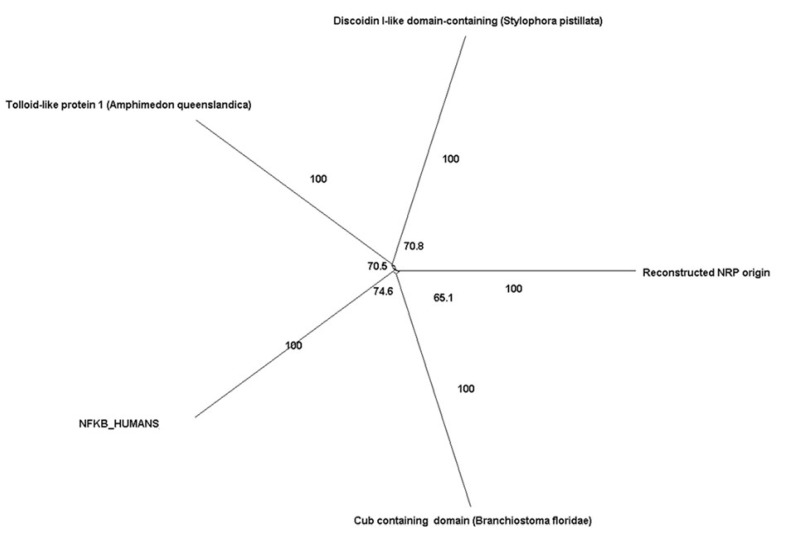
Evolutionary network of Neuropilin family. SplitTrees analysis results suggest that the origin of neuropilin could be more related to Discoidin I-like domains and cub-containing domains than Tollid domains.

The origins of Treg-mediated repressor pathways are diverse. Irrespective of its name, CTLA4 is not related to CTLA1 (Granzyme B), CTLA2, or CTLA3 (Granzyme A). Alternatively, its nearest homolog is CD28. CTLA4 evolved from a protein containing an IGV domain (99.5% and 1 × 10^−17^) (Table 3). LAG3 emerged from an immunoglobulin protein (*Nothobranchius kuhntae)* (99%). The TNFα superfamily contains 19 members that bind to 29 members of the TNF receptor superfamily. Among its members that are known to be employed by Tregs as suppressors are CD40 and TRAIL. We reconstructed a putative ancestral sequence for the TNFα superfamily. Our results indicate that this reconstructed sequence is highly similar to tumor necrosis factor ligand superfamily member 10-like and we identified it in *Lingula unguis* (e-value of 3 × 10^−16^ and 97.97%). Similarly, GITR and CD40R belong to the TNFα receptor family, which contains 27 other receptors. Our results demonstrate that the ancestral sequence of the TNFα receptor family resembles that TNFRSF21-like and it first appeared during the divergence of *Monosiga brevicollis* (Choanoflagellata) with an e-value of 1 × 10^−09^. A20 (TNFAIP3) belongs to the TNFAIPs (Tumor Necrosis Factor, Alpha Induced Protein family). The TNFAIP family mainly includes TNFAIP1, TNFAIP2, TNFAIP3, TNFAIP4, TNFAIP5, TNFAIP6, TNFAIP8, and TNFAIP9. Our results indicate that the ancestral sequence for TNFAIP is likely to be TNFAIP3 like, with a Blastp value of 1 × 10^−40^ and HHsearch probability of 99.8%. Neuropilin 1 (Nrp-1) belongs to the Neuropilin family, which includes one another known member (i.e., Nrp-2) [42]. We identified both Nrp1 and Nrp2 in Cnidaria. This indicates that the origin of the Neuropilin predates Cnidaria divergence. Furthermore, ancestral sequence reconstruction showed that the ancestral sequence of Neuropilin is likely to have contained a cub domain (99%) or a Discoidin I domain (99%) according to HHsearch. The Blastp value indicates that the origin of nearest homolog for Neuropilin family of proteins is Tolloid-like protein 1 in *Amphimedon queenslandica*, with an E-value of 6 × 10^−20^. However, evolutionary network analysis of the Neuropilins does not support the Tolloid-like protein origin hypothesis (Figure 5). Our pheylogentic network analysis indicate that neuropilin origin could be more linked to Discoidin I-like domains, and cub-containing domains than Tolloid domains. GZM B (CTLA1) belongs to the Granzyme family, which is a large family consisting of 11 members, interestingly, only four granzymes appear in human genomes (i.e., A, B, K and M) [43]. Granzymes could have diverged from Trypsin (1 × 10^−38^) (100%), Melanization protease 2 × 10^−33^, or Corin 3 × 10^−32^ [44] (Table 3). CD73 belongs to the 5′nucleotidase family. Seven members of the 5′-nucleotidases have been characterized (i.e., NT5C1A, NT5C1B, NT5C2, NT5C3, NT5C3L, NT5E and NT5M). In agreement with earlier reports, we identified a homolog for the ancestral sequence of CD73 in *Limimaricola hongkongensis* bacteria, with an e-value of 4 × 10^−124^ and HHsesarch value of 100% [45]. Additionally, based on the presence of homologs to this sequence in Algae (5 -nucleotidase [Chlorella sorokiniana], E-value < 4 × 10^−59^) as well as in Fungi (5’-nucleotidase [Xylariaceae sp. FL0016], E-value < 1 × 10^−96^), it could be inferred that the ancestral sequence for 5′nucleotidases have been present in the Last Universal Common Ancestor (LUCA). CD39 belongs to the E-NTPDase family (i.e., ectonucleotidases), which has eight members (NTPDase1, 2, 3, 4, 5, 6, 7 and 8) [46]. The ancestral sequence of Ectonucleotidase seems to be similar to GDA1/CD39 nucleoside phosphatase in Algae (*Helicosporidium* sp. ATCC 50920) (e-value of 7 × 10^−61^). BATF3 belongs to the p21 family, which in turn consists of two more members namely BATF1 and BATF2. The nearest homolog of the reconstructed ancestral for the BATF family is jun dimerization protein 2-like gene that first appeared in Cnidaria *Actinia tenebrosa* (evalue 4 × 10^−06^). The origin of ICER seems to be similar to that of bZIP transcription factor domain containing protein in *Acanthamoeba castellanii* with e value of 7 × 10^−10^ and HHsesarch value 100%. There are five different NFAT members NFATc1, NFATc2, NFATc3, NFATc4, and NFAT5 [44]. The origin of the NFAT seems to be similar to that of the NFATc4 gene, which first appeared in sponges (Table 3). THe Cbl family of ubiquitin ligases consists of three members, namely c-Cbl, Cbl-b and Cbl-c. The nearest homolog to this family is E3 ubiquitin ligase Cbl TKB (tyrosine kinase binding domain) in *Salpingoeca rosetta* (2 × 10^−101^). In vertebrates, the gene family of PPAR consisted of PPAR*α*, PPAR*β* (also called PPARb/d or PPAR*δ*), and PPAR*γ* [47]. The origin of the PPAR family could be related to retinoic acid receptor RXR-alpha-B isoform X1 (*Nematostella vectensis*) (9 × 10^−40^), steroid hormone receptor ERR2-like isoform X2 (*Acropora digitifera*) (1 × 10^−34^), or Dynein heavy chain 6, (*axonemal Exaiptasia diaphana*) (1 × 10^−34^). 

### 3.2. Positive Selection Analysis Indicates That Tregs Employed Both Highly Conserved and Rapidly Evolving Mechanisms 

We investigated positive selection among Treg-mediated suppression pathways (Table 4). We employed PAML for computation of global ω value. Our results show that there are various suppressors employed by Tregs that evolved under strict conservation, mechanisms including CTLA-4 (0.42, *p*-value < 0.0005), as well as the CD40L and its receptors, in addition to TRAIL, GZM, CD73, and GITR. On the other hand, several suppressors employed by Tregs were subjected to strong positive selection, including TGFβ1 (1.46, *p*-value < 0.00001), in addition to LAG3, CD39, NFAT, and Cbl-b (Table 4). 

## 4. Discussion

The evolution of the Treg-mediated suppression function is diverse. The Treg suppression mechanism utilizes direct pathways such as the production of cytokines, inducing apoptosis, regulation of transcription factors in responder cells, regulating ADP/ATP by CD39, and the suppression of calcium signaling. It also uses indirect pathways such as CTLA4 (Table 1). CD39 belongs to the ancient family ectonucleotidases that could have originated from CD39 nucleoside phosphatase in *Helicosporidium sp. ATCC 50920*. Although CD39 seems to have evolved under positive selection ω = 1.45 *p*-value < 0.00001) (Table 4), its ectonucleotidase activity seems to be conserved between invertebrates and mammals^25^. Conversely, CTLA4 seems to be only conserved in vertebrates (ω = 0.42, <0.0005). CTLA4 competes with CD28 for binding to CD80/CD86, and thereby prevents conventional T cell activation. Interestingly, fish CTLA4 and CD28 display homolog structural motifs that are specifically involved in mammalian CTLA4 functionality, supporting the conservation of function hypothesis [48]. The oldest component of this pathway is CD86, which first appeared in Arthropoda, while LFA1 first emerged in Tunicate (Figure 2). Additionally, CTLA4 can suppress conventional T cells by increasing the expression of IDO or reducing the GCLC and GSS expressions. Interestingly, IDO, GCLC and GSS first emerged in sponges. Experimental evidence supports conservation of function for GSS and GCLC, but not IDO (Figure 3) [49]. Taken together, these observations suggest that Tregs employed a versatile set of tools that was not constrained by vertebrate-based homologs.

An important question about the evolution of the suppressive mechanisms utilized by Tregs is the exact time at which Tregs (CD4+ CD25+) came into existence in relation to conventional T cells (CD4+ CD25−). Of particular interest is the Th17/Treg axis, which is critical to the development of an autoimmune response. Th17 and Tregs share a large part of their transcriptome. Previously, RORγt and FoxP3 have been shown to function distinctively as master regulators of Th17 cells and Tregs, respectively. Interestingly, it is now becoming clearer that the landscape of Th17-Tregs axis is more dynamic. Several new populations have been discovered that express one or both transcription factors. These populations include Tr1, RORγt^+^FoxP3^+^IL17^-^ and RORγt^+^FoxP3^+^IL17^+^ cells [8]. It is already known that both Th17 and Tregs belong to the CD4^+^Th group, which first appeared in vertebrates. However, during thymic development, Tregs experience a delay in thymic export, suggesting a difference in timing between Th17 and Treg differentiation hinting at a difference in emergence timing. Our results show that GATA3, the main transcription factor of Th2 cells, has homologs as ancient as Cnidaria (Figure 2). Tbet, the master regulator of Th1 cells, first existed in Spiralia (i.e., Mollusca). RORγt first diverged in Tunicates, and in particular in *Styela clava* (e value < 1 × 10^−22^). Evidence of functional conversation exists for Tbet, IRF4 and GATA3, but not for RORγt (Figure 3). FoxP3 belongs to the FoxP family, which in turn belongs to the Fox superfamily. Fox family origination seems to have taken place during Opisthokonta divergence [50]. However, FoxP3 emergence was estimated to have occurred during early vertebrates’ divergence in conjunction with Treg emergence. Treg-like cells have been found in chicken and zebrafish [51,52]. However, evidence of the existence of CD4^+^ T cells in early fish (e.g., elephant shark) is still lacking. It has been claimed that Th1 cells could be found in cartilaginous fish. However, the existence of Tregs and Th2 cells in elephant sharks is still controversial [53,54]. Furthermore, designating FoxP3 as the sole master regulator of Tregs and as the single piece of evidence for Treg existence could be misleading, as its presence in a given species does not strictly imply that FoxP3 was performing a function analogous to what it performs in higher vertebrates. Thus, the existence of FoxP3 in lower vertebrates such as elephant sharks does not guarantee the existence of Tregs. Therefore, a detailed experimental analysis of the emergence of different CD4^+^ Th subsets in early fish species is still needed.

The mechanism of suppression of immune cells in invertebrates and lampreys is intriguing. In agreement with [55,56] we found that TGFβ2 is expressed in lampreys. It was shown that TGFβ plays an important role in lamprey developmental metamorphosis. Importantly, it has recently been demonstrated that TGFβ2 plays an essential role in regulating the innate immune response by mounting a rapid upregulation in response to lipopolysaccharide stimulation, as well as inhibiting activated leukocytes. Some evidence supports a degree of functional similarity between TGFβ in vertebrates and invertebrates, as it has been shown that the addition of exogenous TGFβ suppresses immune response in Cnidarians [57]. Similarly, we identified the IL6Rβ (GP130) homolog in lampreys, Tunicates, and Spiralia. IL6RB seems to have diverged before IL6 [24]. Additionally, GP130 has been shown to form a complex within the IL6R family, and with IL6RA to form IL6R, IL11R, IL27R, CNTF1R, CNTF2R, and OSMR. However, the absence of compatible receptor chains could indicate that, at least in Tunicates, GP130 is likely to function in a homodimeric fashion to perform an immune-related function in agreement with the principle of ancient receptors promiscuity [25,58]. We localized TRAIL (TNFSF10) in lampreys, Spiralia and Cnidaria. Recently it has been shown that TRAIL induces apoptosis in invading pathogens in blood clam (*Tegillarca granosa*) [59]. This function provides a hint as to how the immune system in jawless vertebrates and invertebrates could constitute a link between the suppression mechanisms of the innate immune system and that of the more novel adaptive immune system. Along the same line of evidence, lampreys possess an ICER protein that is capable of regulating the NFAT pathway, albeit without proof of the functional conservation of ICER in lampreys (Figure 3). Furthermore, the Calcineurin pathways seem to be conserved in jawless vertebrates. Lampreys have two main types of adaptive immune cells that are analogous to T cells (VLRA and VLRC) [60]. Interestingly, FoxP3 does not seem to appear in lampreys, suggesting that lampreys lack a FoxP3+ Tregs. It is important to note that lamprey genome sequencing databases suffer from a degree of bias because of the high GC content, leading to assembly fragmentation [61,62]. Further advancement in the field of whole-genome sequencing could prove vital to shedding light on the evolution of suppression mechanisms in lampreys. Taken together, our results hint that lampreys could possess a Treg-like cell; however, its function is not mediated by FoxP3.

The versatility of functional suppressor mechanisms is not confined to Tregs, but could also be expanded to responder cells. The CD40-CD40L pathway, which is known to be used by CD4+ CD25- T cells to resist Treg suppression, evolved under significant ancient functional conservation (*p*-values = 0.00001 and 0.00002, respectively). A20, which is used by Tregs to prevent DCs from inhibiting Tregs, does seem to have evolved under neutral selection, following Kimura’s hypothesis [63] (Table 4). In summary, adaptive immune cells seem to have adopted mixtures of mechanisms to both suppress and evade suppression.

Our investigation supports the argument of a convergent origin of regulation of CD4+ helper T cells. We found that CTLA4 and LAG3 seem to have diverged from Ig-like proteins. On the other hand, TRAIL and CD40L seem to have diverged from TNFRS10. Interestingly, GITR and CD40R diverged from the TNFR superfamily, highlighting the role of the TNF family and its receptors in CD4+ T cell regulation. This is further supported by the emergence of A20 from a TNF α-induced family in sponges (Table 3). However, other protein families, such as CUB and trypsin (99%)-containing domains, appear to have contributed to Treg suppressor function. CD73 belongs to the ancient Ecto-5’-nucleotidase family, which could have risen from *Limimaricola hongkongensis. Drosophila* has two CD73 homologs that regulate the axis of pro-inflammatory ATP/anti-inflammatory adenosine using a similar mechanism to that of Tregs. The reconstructed ancestral sequence of ICER seems to be homologous to the bZIP transcription factor domain-containing protein in amoeba (*Acanthamoeba castellanii*) with an e-value of 7 × 10^−10^ (Table 2). Our results suggest that the suppression mechanisms of Tregs do not have a single origin. Conversely, Treg suppression mechanisms’ proteins seem to belong to different protein families.

## 5. Conclusions

Our understanding of the evolutionary history of Tregs is by no means complete. There are competing theories about the process of neo-/sub-functionalization of duplicated genes. It is unclear if Treg evolution followed a conventional or a DCC pattern or a mixture of both methods. These observations imply that the conservation of function is a multi-factorial process. Thus, it could be controlled by other factors in addition to the conservation of sequences among various species. This, in turn, paints a complex picture of how Tregs acquired their versatile suppression techniques and inherently supports a convergent origin for Tregs’ comprehensive abilities. Our findings suggest that Tregs have repurposed a limited number of ancient suppression mechanisms that are probably connected to invertebrates. Tregs used various techniques that are conserved in higher vertebrates. These techniques could be more suitable for the needs of the adaptive immune system. These findings suggest that Treg cell evolution was a selective process in which Tregs chose pathways from vertebrates and invertebrates that best fit their purpose.

## Figures and Tables

**Table 1:** Identified suppression mechanisms utilized by Tregs.

	Pathway	Genes
Indirect pathway	Inhibit DC	CTLA4, CD28, CD80, CD86, LFAI-1, A20, CD40-CD40L, neuropilin-1 and LAG3
Direct pathways	Produce Suppressive cytokines	TGFβ, IL10 and IL35
	Consumption of cytokines	IL2, IL2Rα, IL2Rβ and IL2Rγ
	Induction of apoptosis	TRAIL, CD3, CD46, CD25 and BIM
	Regulation of TF	IRF4, GATA3, FoxP3 and RORγt
	ATP and ADP regulation	CD73, CD39, APRT, A2A receptors, and P2RY11
	cAMP/NFAT regulation	IL4, ICER, PPRγ, p21SNFT, GITR and CBLB
	Calcium signaling	NFκB, PPP3CA, PPP3CB, PPP3CC, IKKα, IKKβ, IKKγ, IκB IKBA, and p65

**Table 2:** Experimentally validated conservation of function for Treg suppressor pathways.

	Gene	Conserved Function between Vertebrates and Invertebrates	Conserved Function within Vertebrates
1	CTLA4	No evidence for conservation of function	PMID: 16547256
2	CD28	No evidence for conservation of function	PMID: 16547256
3	CD80	No evidence for conservation of function	PMID: 30107249 PMID: 19535623
4	CD86	No evidence for conservation of function	PMID: 30107249 PMID: 19535623
5	Lfa1	No evidence for conservation of function	
6	IDO1	No evidence for conservation of function	
7	GSS	Conserved function in Drosophila PMID: 18779381	PMID: 21802407
8	GCLC	Conserved function in Drosophila PMID: 19036725	PMID: 28158580
9	CD40	No evidence for conservation of function	PMID: 35667467
10	CD40L	No evidence for conservation of function	PMID: 35667467
11	A20	No evidence for conservation of function	PMID: 33154446
12	Neuropilin	No evidence for conservation of function	
13	Lag3	No evidence for conservation of function	PMID: 16951357
14	Tgfb1	No evidence for conservation of function	
15	Tgfb2	No evidence for conservation of function	
16	Il10	No evidence for conservation of function	PMID: 25416810
17	IL10RA	No evidence for conservation of function	PMID: 25416810
18	IL10RB	No evidence for conservation of function	No evidence for conservation of function
19	IL27B(IL35)	No evidence for conservation of function	PMID: 30590066
20	IL12A	No evidence for conservation of function	PMID: 30204772 (similar)
21	GP130	Conserved in Drosophila PMID: 14504285	PMID: 18687405
22	IL12RB	No evidence for conservation of function	No evidence for conservation of function
23	IL2	No evidence for conservation of function	PMID: 30093902
24	IL2RA	No evidence for conservation of function	PMID: 30093902
25	IL2RB	No evidence for conservation of function	PMID: 35218892
26	IL2RG	No evidence for conservation of function	PMID: 35218892
27	Trail	Seemed to be homologs with Drosophila gene Eiger, PMID: 12065414. Evidence for conserved function of inducing apoptosis.	PMID: 33483333
28	Cd3g	No evidence for conservation of function	PMID: 19744563
29	Cd3d	No evidence for conservation of function	PMID: 19744563
30	Cd3e	No evidence for conservation of function	PMID: 19744563
31	Cd46	No evidence for conservation of function	PMID: 25452563
32	Gzm	No evidence for conservation of function	PMID: 31736981
33	Bim (Bcl2l11)	No evidence for conservation of function	No evidence for conservation of function
34	Tbet	Some function conserved in ectoderms	PMID: 25016582
35	Irf4	Conserved function not proven	PMID: 19638535
36	Gata3	Conserved function not proven	PMID: 19638535 PMID: 12100886
37	Rorgt	Conserved function not proven	PMID: 16990136
38	Cd39	Conserved function in Drosophila and even in plants	PMID: 9676430
39	Cd73	Conserved function in Drosophila	PMID: 21996016
40	A2ar	Seems to have a conserved in Drosophila	PMID: 32108870
41	P2ry11	No evidence for conservation of function	
42	Il4	No evidence for conservation of function	PMID: 32641385
43	Il4r	No evidence for conservation of function	
44	Icer	Well conserved in invertebrates on a functional level	PMID: 19434522
45	Nfat1	No evidence for conservation of function	
46	Nfat4	No evidence of conserved function	
47	Pprγ	No evidence of conserved function	
48	P2 1sfnt (BATF3)	No evidence of conserved function	
49	Girt	No evidence for conservation of function	
50	Cblb	Seems Conserved function in both C.elegans and drosophila PMID: 10542134	PMID 16227975
51	Nfkβ	Conserved function in Drosophila	PMID: 20457557
52	Ppp3ca	No evidence of conserved function	
53	Ppp3cb	No evidence of conserved function	
54	Ppp3cc	No evidence of conserved function	
55	Ikka	No evidence of conserved function	
56	Ikkβ	Conserved function	PMID: 34452932
57	Ikkγ	Conserved function	PMID: 11017107
58	Ikb	No evidence for conservation of function	No evidence of conserved function
59	P65	No evidence of conserved function	

**Table 3:** Putative origins for Treg-mediated pathways.

Molecule	Main Family	Putative Origin	Blastp E-Value	HHsearch Probability (%)
CTLA4	Immunoglobulin superfamily	IGv domain-containing protein (*Callorhinchus milii*)	1 × 10^−17^	99.5
LAG3		IG-like proetin (*Nothobranchius kuhntae*)	NA	99
CD40L	Tumor necrosis factor (TNF) superfamily	Tumor necrosis factor ligand superfamily member 10-like (*Lingula unguis*)	3 × 10^−16^	97.9
TRAIL				
GITR CD40R	Tumor Necrosis Factor Receptor Superfamily	TNFRSF21-like (*Monosiga brevicollis*) (Choanoflagellata)	1 × 10^−09^	NA
A20 (TNFAIP3)	Tumor Necrosis Factor, α Induced Protein family	Tumor necrosis factor α-induced protein 3like (*Amphimedon queenslandica*)	1 × 10^−40^	99.8
Neuropilin-1	Neuropilin family	Tolloid-like protein 1 (*Amphimedon queenslandica*)	6 × 10^−20^	NA
		Cub-containing domain (*Branchiostoma floridae*)	NA	100
		Discoidin I-like domain-containing (*Stylophora pistillata*)	NA	99
GZM B (CTLA1)	Granzymes	Trypsin (*Asbolus verrucosus*)	1 × 10^−38^	100
		Melanization protease 1	2 × 10^−33^	NA
		Corin, isoform C (*Drosophila melanogaster*)	3 × 10^−32^	NA
CD73 (NT5E)	Ecto-5’-nucleotidase	5’-nucleotidase/apyrase family protein (*Limimaricola hongkongensis*)	4 × 10^−124^	100
CD39	E-NTPDase family of ectonucleotidases	GDA1/CD39 nucleoside phosphatase (*Helicosporidium sp. ATCC 50920*)	7 × 10^−61^	NA
BATF (p21SNFT)	Basic leucine zipper transcriptional factor ATF-like	jun dimerization protein 2-like (*Actinia tenebrosa*)	4 × 10^−06^	NA
ICER	Unknown	bZIP transcription factor domain containing protein (*Acanthamoeba castellanii*)	7 × 10^−10^	100
NFAT	NFATs	NFATc4 (*Amphimedon queenslandica*)	5 × 10^−23^	NA
Cbl-b	ubiquitin ligase	E3 ubiquitin ligase Cbl TKB (*Salpingoeca rosetta*) (Choanoflagellates)	2 × 10^−101^	NA
PPRγ	5 hydroxyicosatetraenoic acid and 5-oxo- eicosatetraenoic acid family	retinoic acid receptor RXR-alpha-B isoform X1 (*Nematostella vectensis*)	9 × 10^−40^	NA
		steroid hormone receptor ERR2-like isoform X2 *(Acropora digitifera)*	1 × 10^−34^	NA
		*Axonema* Dynein heavy chain 6 *(Exaiptasia diaphana)*	1 × 10^−34^	NA

**Table 4:** Positive selection analysis of genes likely to be used by Tregs as suppressors.

Molecule	Global (ω) Value	*p*-Value
CTLA4	0.42	<0.0005
CD40	0.27	<0.00001
CD40L	0.39	<0.00002
A20 (TNFAIP3)	0.96	not significant at *p* < 0.05.
Neuropilin-1	1.13	not significant at *p* < 0.05.
LAG3	1.50	<0.00001
TGFβ1	1.46	<0.00001.
TRAIL	0.44	<0.00006
GZM	0.31	<0.00001
CD39	1.45	<0.00001
CD73	0.48	<0.00001
ICER	0.63	<0.01
NFAT	1.88	<0.00001
PPRγ	0.61	not significant at *p* < 0.05.
p21SNFT	0.66	not significant at *p* < 0.05.
GITR	0.50	<0.0001
Cbl-b	1.38	<0.00001

## Data Availability

The data used in this manuscript are available upon request.
